# Tibial lengthening with LRS fixator in a pediatric patient with congenital anterolateral bowing: A case report

**DOI:** 10.1016/j.ijscr.2025.111722

**Published:** 2025-07-25

**Authors:** Rakesh Kumar Yadav, Gaurav Parajulee, Aarya Dahal

**Affiliations:** a Department of Orthopedics and Trauma Surgery, Tree Top Hospital, Maldives; b Department of Orthopedics, Janaki Medical College and Teaching Hospital, Nepal

**Keywords:** Congenital tibial bowing, Limb length discrepancy, LRS fixator, External fixation, Pediatric limb lengthening, Case report

## Abstract

**Introduction and importance:**

Congenital anterolateral bowing of the tibia is a rare orthopedic condition that can lead to significant limb length discrepancy (LLD) during growth. Gradual limb lengthening with external fixation remains a reliable method to achieve limb equalization. LRS fixator offers a minimally invasive and adjustable treatment option for pediatric patients considering the growth potential.

**Case presentation:**

We present the case of a 9-year-old boy with a known case of right-sided anterolateral bowing of the tibia, who presented with a 3 cm limb length discrepancy. The patient underwent minimally invasive corticotomy and tibial lengthening using a Limb Reconstruction System (LRS) external fixator. The postoperative period was free of complications with intended lengthening achieved.

**Clinical discussion:**

Preoperative planning, growth potential and radiological assessment, and precise corticotomy are essential in limb lengthening procedures in pediatric patients. In this case, Paley multiplier method predicted a final discrepancy of about 6 cm at maturity which led to a decision to lengthen 3 cm at this stage and the External Fixator Index (EFI) was 50 days/cm, corresponding to five months of non weight bearing External Fixator use. No complications such as neurovascular compromise, pin tract infection, or joint stiffness were observed post-implant removal or during ROM exercises for rehabilitation.

**Conclusion:**

Tibial lengthening using LRS is an effective method for managing moderate LLD in pediatric patients if the growth potential is adequately kept in account. Early intervention, meticulous surgical technique, and close follow-up are key factors for a successful outcome.

## Highlights

• Congenital anterolateral tibial bowing can cause in progressive limb length discrepancy in pediatric patients.
• Gradual tibial lengthening was done with a monolateral LRS external fixator after precise preoperative planning.
• Paley Multiplier Method was used to estimate final discrepancy and guide lengthening plan.
• 3 cm tibia lengthening was achieved with an External Fixator Index of 50 days/cm and no complications.
• Case highlights safe application of distraction osteogenesis in pediatric patients with open growth plates.

## 1 Introduction

Congenital anterolateral bowing of the tibia is a rare orthopedic condition often identified at birth. While it can be an isolated anomaly, it's frequently associated with neurofibromatosis type 1 (NF1) and may progress to congenital pseudarthrosis of the tibia (CPT) [1]. However, not all cases lead to CPT. [2]. These deformities may progress to tibial shortening, leading to LLD and gait abnormalities. Limb lengthening with external fixation, including devices like the LRS, offers a minimally invasive and adjustable treatment option**.** However, consideration of growth potential while solving the discrepancy plays a pivotal role.

Our case has been reported in line with SCARE 2025 criteria. [3]

## 2 Case presentation

**Fig. 1:**
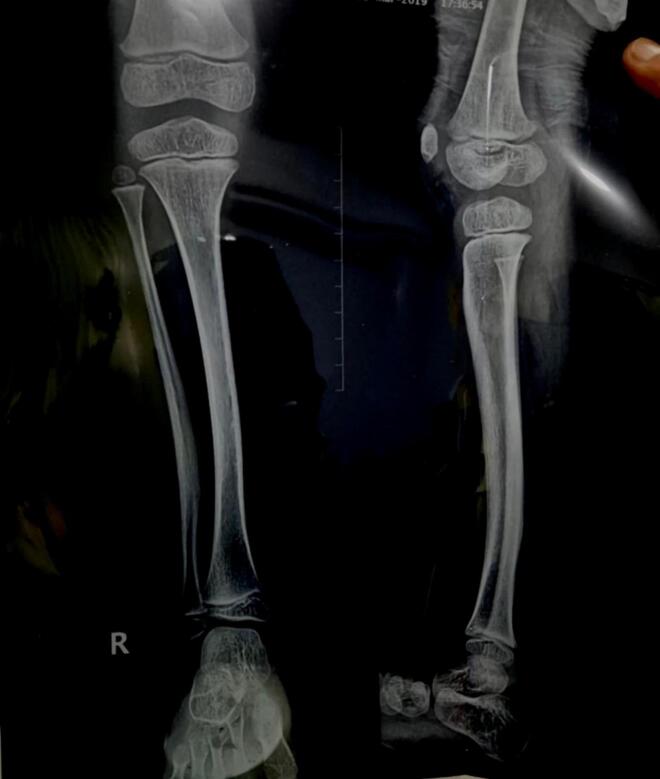
X-ray of Right leg (anteroposterior and lateral views) showing anterolateral bowing of the right tibial diaphysis. The X-ray dates back to when the patient was 3 years old.

A 9-year old boy was brought to our institution with a right lower limb length discrepancy of approximately 3 cm. The patient had a known history of congenital anterolateral bowing of the right tibia, diagnosed during early childhood.(Fig. 1) The patient, being a school-going child, had bothersome experience due to the discrepancy and bowing. There were no other members in the family or close relatives who complained of the similar deformity. The patient had no significant past medical history for which he had to be hospitalized and had not undergone any surgeries in the past. The parents did not give any history of drug allergy.

Clinical examination revealed stable hips, no significant deformities in the contralateral limb, and an otherwise normal gait pattern. No signs of inflammation, no spine deformities or disproportionate growth pattern in the child was observed. On local examination of the right leg of the child, the range of movement at ankle joint was not restricted and the gait was nearly optimal in character. Conservative management with a shoe raise had been advised for the next three months of their first visit.

After three months of being on a shoe raise, Surgical limb lengthening using an LRS external fixator was indicated due to a progressive and symptomatic 3 cm limb length discrepancy in our 9-year-old child with congenital anterolateral bowing of the tibia. Given the child's age and remaining growth potential, gradual lengthening with external fixation in a minimally invasive technique was deemed appropriate to achieve limb equalization and prevent long-term gait abnormalities and pelvic imbalance.

### 2.1 Pre Operative planning

A CT scan was obtained to assess limb length and bone quality and alignment. CT also aids in planning of corticotomy level and lengthening amount. Marking of pin insertion sites under fluoroscopy was done and the patient party was counseled and an informed consent was taken.

### 2.2 Anesthesia and positioning

General Anesthesia was given to the patient and the patient was placed in supine position with a bolster under the knee. Aseptic precaution was taken and draping of the limb was done.

### 2.3 LRS Fixator frame application

The levels of pin insertion and corticotomy were marked which was followed by insertion of three Schanz pins, perpendicular to the mechanical axis of tibia, proximally and distally to the planned corticotomy site. Drill-sleeve technique was used to prevent thermal necrosis. Then, the 30 mm LRS rail was attached to the pins and the fixator was tightened.

### 2.4 Corticotomy and completion of fixator assembly

A 2-3 cm incision was given on the skin over the corticotomy site. A percutaneous mid-diaphyseal corticotomy of the right tibia was then carried out. A 1 cm segment of the fibula was excised via a lateral approach following subperiosteal dissection. Schanz pins were inserted proximal and distal to the osteotomy site, and a Limb Reconstruction System (LRS) external fixator was applied using a 30 mm rail system and two clamps. Layered closure was performed to complete the procedure. The alignment and stability was checked under fluoroscopy.

### 2.5 Post Operative period and Discharge

**Fig. 2:**
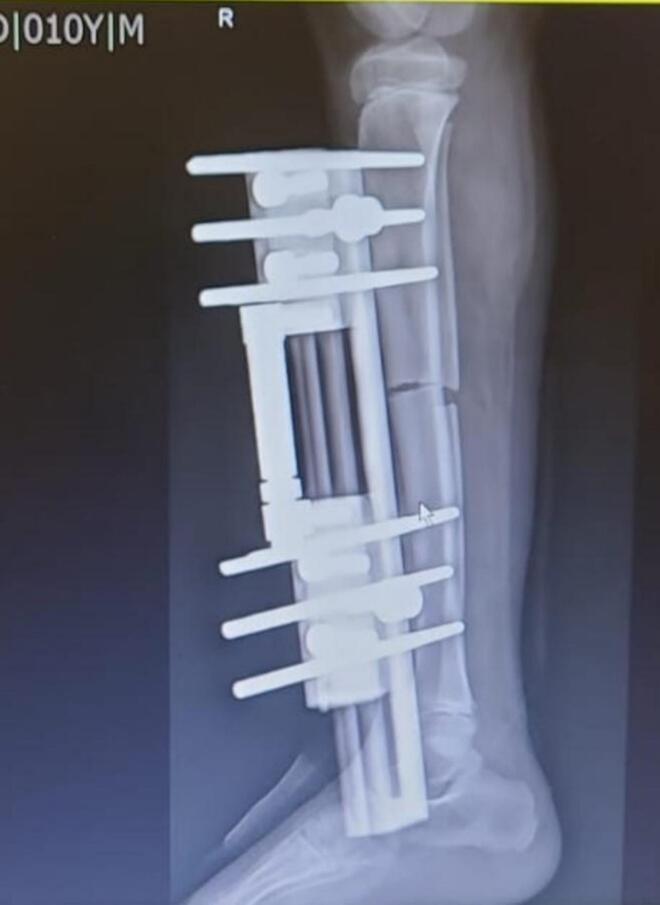
Xray Right leg (lateral view)- first post-operative day showing LRS fixator inserted with three Schanz pins each on either side of the mid diaphyseal corticotomy site of right tibia.

Post operative day one was comfortable for the patient. Serial X-rays were taken of the operated leg. (Fig. 2) He had no toe swelling and sensation was intact. The epidural was tapered and i.v. paracetamol was commenced. The patient was shifted to the ward and the parents were taught about starting distraction from seven days. The first five post operative days were latency period when the patient was put in rest to allow early callus formation. On fifth post-operative day, physiotherapy of the patient with nonweight bearing walker as tolerated was started and the patient was discharged.

### 2.6 Distraction phase and followup

In growing children, the intended amount of limb lengthening must consider both the current discrepancy and the predicted future discrepancy at skeletal maturity. In our case, the child had a 3 cm limb length discrepancy. Using the Paley Multiplier Method, which estimates future limb length discrepancy based on chronological age and current measurements, we calculated that the untreated discrepancy would likely progress to approximately 6–6.5 cm at maturity. Given that our patient was 9 years old with open growth plates, we opted for a 3 cm lengthening at this stage to address the functional limitation and gait abnormality, while preserving options for future growth modulation (e.g., contralateral epiphysiodesis or additional lengthening) if required.

This approach balances the benefit of early correction with the risk of over-lengthening in a skeletally immature patient. Continuous monitoring of growth and reassessment at puberty will help guide further management.

**Fig. 3:**
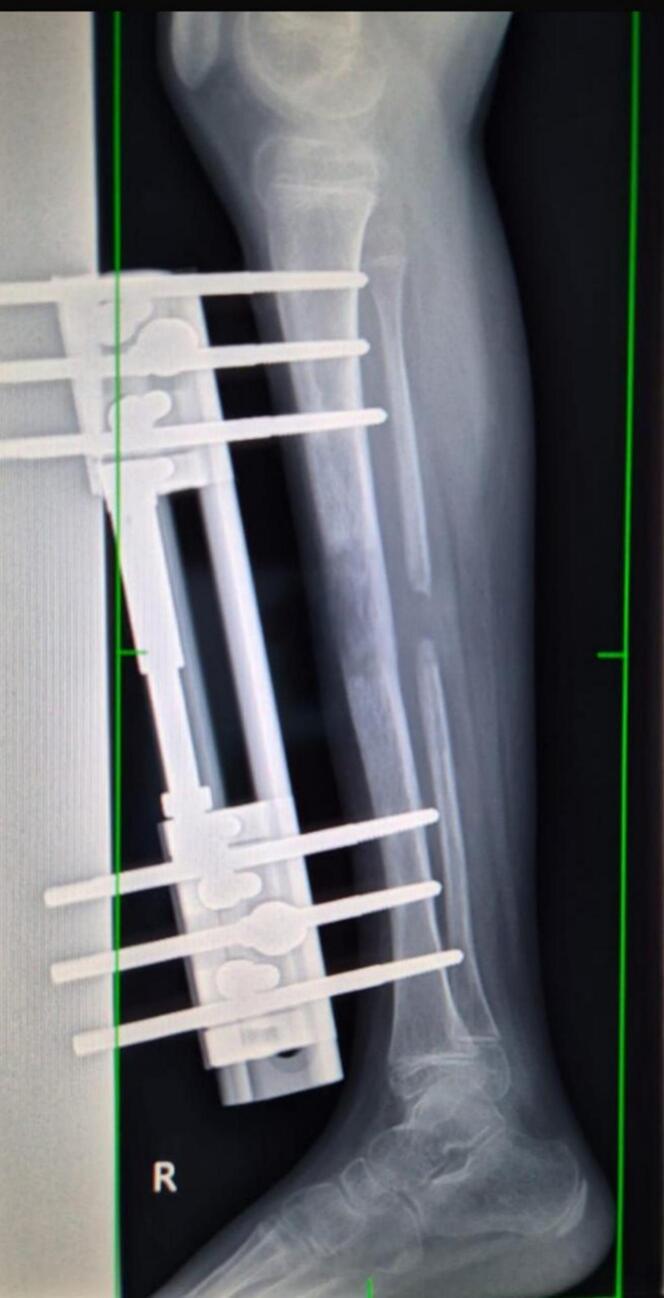
Xray Right leg (lateral view)- 6 weeks of distraction showing callus formation and bone lengthening of right tibia.

Distraction Phase was initiated on postoperative day seven at 1 mm/day in four increments of 0.25 mm each day. Serial X-rays demonstrated satisfactory callus formation by the sixth week of distraction. **(**Fig. 3**)**. The lengthening phase continued for 6 weeks, at which point clinical limb length equalization was achieved. The patient remained non-weight bearing during this period and was closely monitored for signs of neurovascular compromise, pin tract infection, and joint stiffness. At two months postoperatively, both limbs were clinically equal in length, and full range of motion of the knee was preserved.

### 2.7 Implant removal

**Fig. 4:**
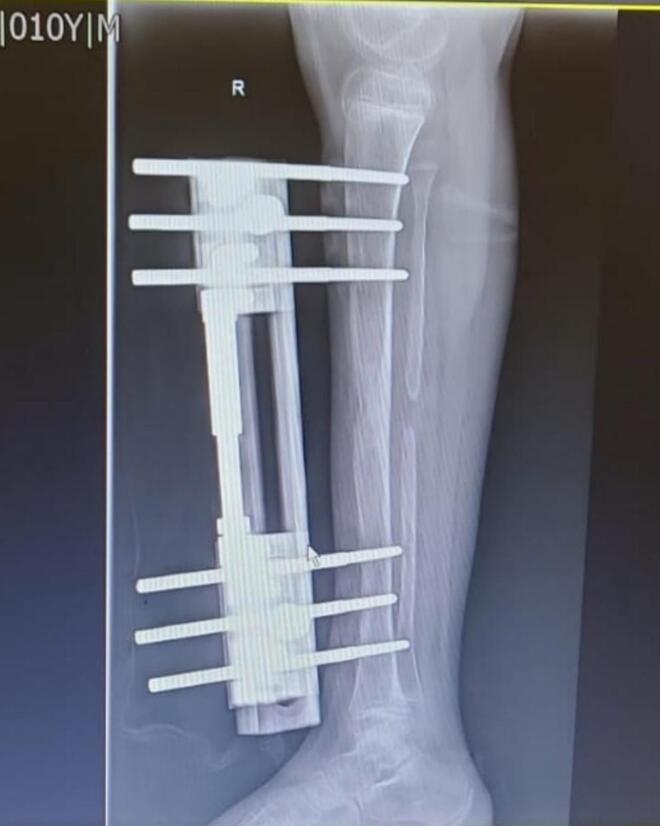
Xray Right Leg (Lateral view) taken just before implant removal confirming 3 cm increase in tibial length and clinical limb equalization.

**Fig. 5:**
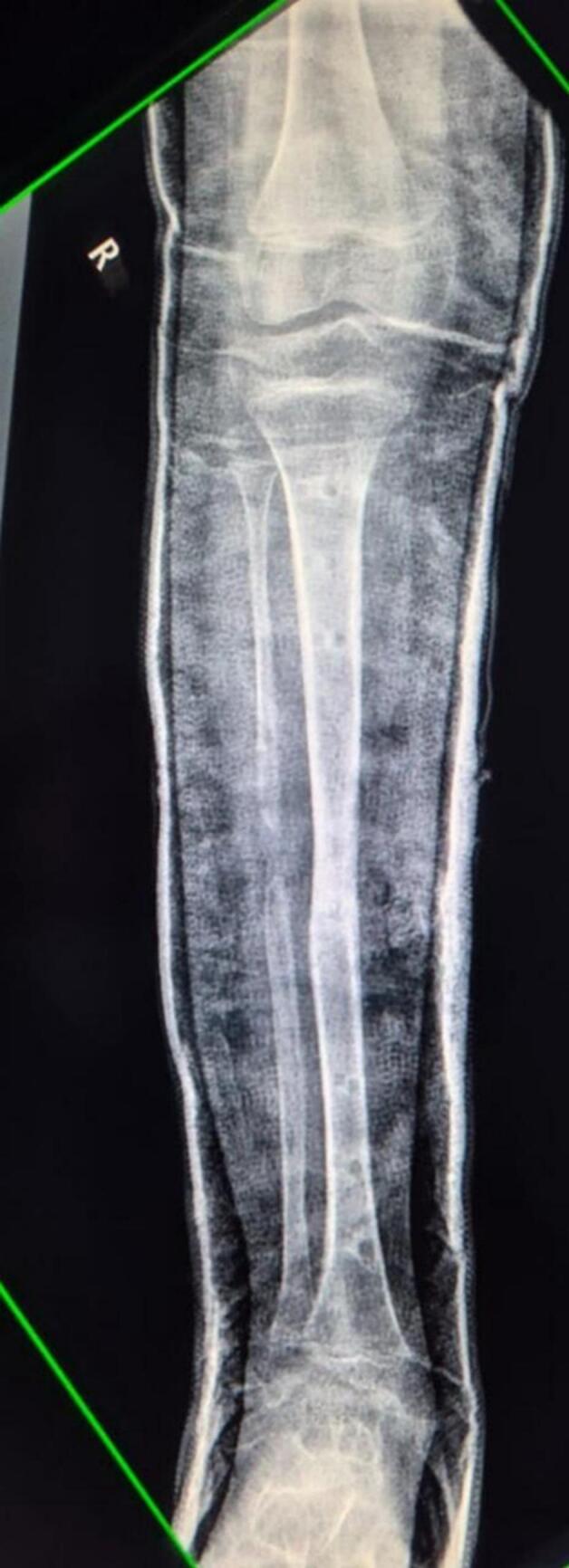
Xray Right leg (AP view) with above knee cast insitu for stabilization post LRS fixator removal.

Pre-implant removal radiograph was taken. (Fig. 4**)**. Implant removal was performed after 5 months of operation under sterile conditions. External Fixator Index (EFI) was 50 days/cm, corresponding to five months of External Fixator use. Intraoperative fluoroscopy confirmed radiological union at the osteotomy site. An above-knee cast was applied for additional stabilization, and the patient was discharged the following day in stable condition following an Xray. (Fig. 5) No intraoperative or postoperative complications were observed. Wound remained dry with no soakage.

### 2.8 Followup

**Fig. 6:**
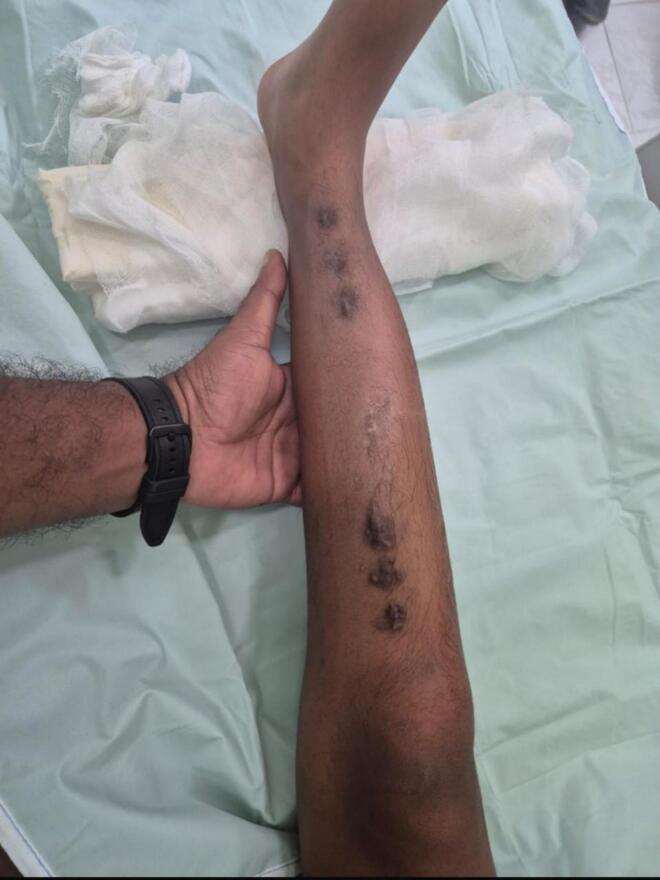
Six Schanz pin site scars post LRS fixator removal with healing; bowing corrected.

Subsequent followups at 3, 6, and 9 months of discharge revealed that the child had an excellent functional and radiological outcomes. Limb length equality was achieved, the patient was able to ambulate independently without discomfort or gaity abnormality and there was no pelvic tilt. There were no signs of complications such as infection, joint stiffness, or deformity recurrence. Schanz pin site scars were healed. **(**Fig. 6**).**

## 3 Discussion

### 3.1 Etiology and natural history

Congenital anterolateral bowing of the tibia is a rare orthopedic condition often identified at birth. While it can be an isolated anomaly, it's frequently associated with neurofibromatosis type 1 (NF1) and may progress to congenital pseudarthrosis of the tibia (CPT) [1]. However, not all cases lead to CPT. Some instances, such as congenital anterolateral bowing with polydactyly, represent distinct entities with favorable prognoses and are not linked to NF1 [2].

The natural course of congenital tibial bowing varies. Posteromedial bowing often demonstrates spontaneous improvement during early childhood, with significant remodeling occurring within the first few years of life [4]. In contrast, anterolateral bowing has a higher risk of progression to CPT, especially when associated with NF1-positive patients [1].

### 3.2 Limb length discrepancy and surgical indications

LLD is a common consequence of congenital tibial bowing. Studies have shown that the degree of initial bowing correlates with the magnitude of LLD at skeletal maturity [5]. While discrepancies under 2 cm may be managed conservatively, surgical intervention is considered when LLD exceeds 2–3 cm or when associated with functional impairment, pelvic tilt or gait disturbance [4,6].

The optimal timing of surgical correction remains a topic of debate. Some authors advocate for delaying lengthening procedures until closer to skeletal maturity to minimize complications and the need for repeat surgeries [7]. Others suggest that early intervention may be beneficial in cases of severe deformity or functional limitation [8].

Performing limb lengthening in a skeletally immature patient requires careful consideration of the open epiphyses and the child's remaining growth potential. In children, overcorrection or premature lengthening can lead to future discrepancies due to ongoing growth. However, delaying surgery in symptomatic children with significant discrepancy may result in progressive pelvic tilt, gait abnormalities, and psychological impact.

In this case, the child was 9 years old with open growth plates and had a projected final discrepancy of approximately 6 cm based on the Paley Multiplier Method. A staged correction strategy was adopted: a 3 cm lengthening was performed to address the current functional limitation, while maintaining growth potential for future modulation via contralateral epiphysiodesis or additional lengthening if needed. Furthermore, younger children have a better remodeling potential, and early correction can prevent long-term musculoskeletal compensations. Throughout the process, we ensured that the physes were not violated during pin placement or corticotomy, minimizing any risk of premature physeal closure.

### 3.3 Surgical techniques and outcomes

Various surgical techniques have been employed to address LLD in the context of congenital tibial bowing. The Ilizarov method, utilizing circular external fixation, has been widely used and allows for simultaneous correction of deformity and lengthening. [9]. However, this method can be associated with complications such as pin tract infections, joint stiffness, and regenerate bone fractures.

**Fig. 7:**
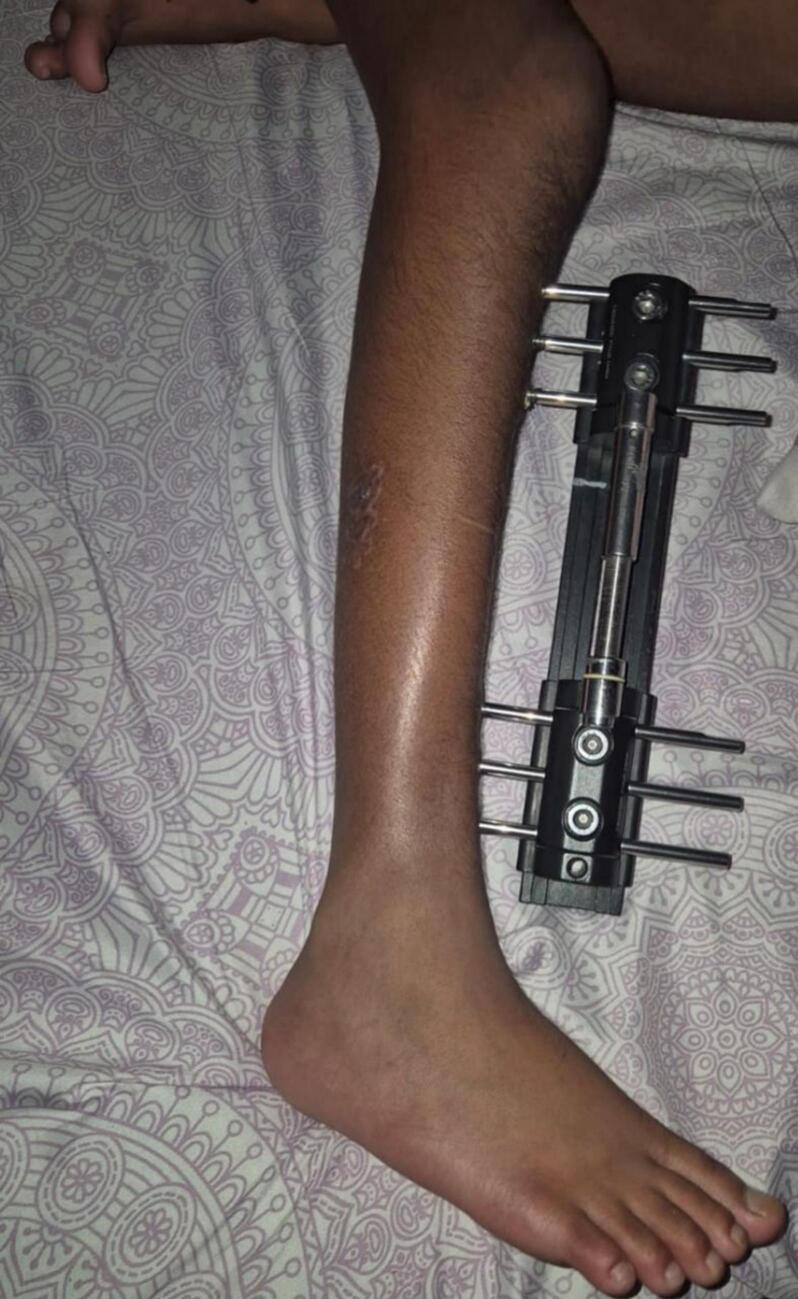
LRS monorail fixator insitu for Right tibial lengthening in a pediatric case of anterolateral bowing and limb length discrepancy; comfortable over ring fixator.

The Limb Reconstruction System (LRS), a monolateral external fixator, offers an alternative approach. Its advantages include ease of application, patient comfort, and effective control over the distraction process. LRS fixator was a better choice for our patient in terms of comfort and easier handling in pediatric setting **(**Fig. 7). While literature specific to LRS in congenital tibial bowing is limited, its efficacy in pediatric limb lengthening is well supported [7].

The LRS fixator offers excellent advantages in terms of earlier weight bearing, shorter hospital stays, fewer complications and better radiological outcomes. [10]

### 3.4 Complications and considerations

Complications associated with limb lengthening procedures include pin tract infections, joint stiffness, neurovascular injury, and malalignment. The risk of complications can be mitigated through meticulous surgical technique, appropriate patient selection, and rigorous postoperative care and followup [7,9].

In cases of congenital anterolateral bowing, careful monitoring is essential due to the potential progression to CPT. Early identification and intervention can prevent pathological fractures and the development of pseudarthrosis [1]. Performing limb lengthening in a skeletally immature patient requires careful consideration of the open epiphyses and the child's remaining growth potential.

In children, overcorrection or premature lengthening can lead to future discrepancies due to ongoing growth and delaying surgery in symptomatic children with significant discrepancy may result in progressive pelvic tilt, gait abnormalities, and psychological impact. These considerations formed the backbone to our treatment aim.

## 4 Conclusion

This case demonstrates that gradual limb lengthening using external fixation is a safe and effective method for correcting moderate limb length discrepancies in children. With careful planning, consideration of the growth potential, precise technique, and close monitoring, excellent functional outcomes can be achieved.

## Author agreement statement

We the undersigned declare that this manuscript is original, has not been published before and is not currently being considered for publication elsewhere.

We confirm that the manuscript has been read and approved by all named authors and that there are no other persons who satisfied the criteria for authorship but are not listed. We further confirm that the order of authors listed in the manuscript has been approved by all of us.

We understand that the Corresponding Author is the sole contact for the Editorial process. He/she is responsible for communicating with the other authors about progress, submissions of revisions and final approval of proofs.

## Patient perspective

“I am satisfied with the treatment I have received. I can walk around with no shoe raise required or without limping.”

## Consent

Written informed consent was obtained from the patient for publication of this case report. A copy of the written consent is available for review by the editor-in-chief of this journal on request.

## Provenance and peer review

Not commissioned, externally peer-reviewed.

## Ethical approval

This is a case report, therefore, it did not require ethical approval from the ethics committee at our institution. Written informed consent was obtained from the patient's parents/legal guardian for publication and any accompanying images.

## Funding

The study did not receive any grant from funding agencies in the public, commercial or not-for-profit sectors.

## Author contribution

**Gaurav Parajulee (GP) and Aarya Dahal (AD) =** Writing manuscript, Concept, referencing.

**Rakesh Kumar Yadav (RKY) =** Operating Orthopedic Surgeon, Study concept, Reviewing.

All the authors read and approved the final manuscript.

## Guarantor

Gaurav Parajulee accepts full responsibility for the work and/or the conduct of the study, had access to the data, and controlled the decision to publish.

## Research Registration Number

N/A.

## Acknowledgments

The authors would like to thank the patient and their family for providing consent and for their cooperation throughout the treatment and follow-up. We also acknowledge the support of the orthopedic team and nursing staff involved in the patient's care.

## Conflict of interest statement

None.

## References

[1] Mastantuoni G., Vitiello R., Caldari E. (2023). Anterolateral congenital tibial bowing: case report. Front. Pediatr..

[2] Lemire E.G. (2007). Congenital anterolateral tibial bowing and polydactyly: a case report. J. Med. Case Rep..

[3] Kerwan A., Al-Jabir A., Mathew G., Sohrabi C., Rashid R., Franchi T., Nicola M., Agha M., Agha R.A. (2025). Revised surgical CAse REport (SCARE) guideline: an update for the age of artificial intelligence. Premier Journal of Science.

[4] Di Gennaro G.L., Peruzzi M., Baroni G. (2020). Deformity progression in congenital posteromedial bowing of the tibia: a report of 44 cases. BMC Musculoskelet. Disord..

[5] Sevencan A., Aykut U.S., Koca K. (2022). Evolution of the angular deformity and limb length discrepancy of congenital posteromedial bowing of the tibia over time. Joint Dis. Relat. Surg..

[6] Paley D. (2002).

[7] Kale S., Bansal A., Sharma K. (2023). Congenital pseudoarthrosis of tibia with anterolateral bowing treated with Ilizarov ring fixator: a case report. Cureus.

[8] Gordon J.E., Nduaguba A., Beckwith T. (2020). Limb lengthening in the treatment of posteromedial bowing of the tibia. J. Child. Orthop..

[9] Laubscher M., Mishra A., Ferreira N. (2024). Results of pediatric tibial lengthening using the PRECICE intramedullary nail. J. Pediatr. Orthop..

[10] Pal C.P., Kumar H., Kumar D., Dinkar K.S., Mittal V., Singh N.K. (2015). Comparative study of the results of compound tibial shaft fractures treated by Ilizarov ring fixators and limb reconstruction system fixators. Chin. J. Traumatol..

